# PD75 Using The Quality-Adjusted Life-Year For Economic Evaluations Of Public Health Interventions: Benefits And Criticisms

**DOI:** 10.1017/S0266462324003301

**Published:** 2025-01-07

**Authors:** Nur Atika

## Abstract

**Introduction:**

The number of economic evaluations of public health intervention is gradually increasing, which corresponds to the increasing interest of policymakers in public health interventions. However, there are some methodological challenges and debates regarding economic evaluations of public health interventions due to the nature and complexity of public health programs. One of the challenges is related to the outcome measure used.

**Methods:**

This narrative review explored the advantages and disadvantages of using the quality-adjusted life-year (QALY) as an outcome measure in economic evaluations of public health interventions.

**Results:**

The QALY is a preferred outcome measure in cost-utility analyses because of its simplicity, clarity, face validity, and ease of application. The QALY provides a measure of overall health and allows for comparability across different cases. However, there are some criticisms of using the QALY in economic evaluations of public health interventions. Many public health programs aim to affect not only health outcomes, but also other aspects such as participation and empowerment. In addition, public health programs might have positive externalities. The QALY is unable to detect minor changes resulting from community-based health interventions and does not capture equity aspects of public health interventions.

**Conclusions:**

It is necessary to establish a common framework for a transparent and consistent decision-making process that accounts for the multidimensional and complex outcomes of public health interventions.

